# Relationship between long non-coding RNA polymorphism and the risk of coronary artery disease: A protocol for systematic review and meta-analysis

**DOI:** 10.1097/MD.0000000000025146

**Published:** 2021-03-26

**Authors:** Bolin Wang, Zhihui Su, Lijun Wan, Tao He

**Affiliations:** a Department of interventional medicine; b Department of orthopedics, People's Hospital of Wuhan University Hanchuan Hospital, Hanchuan People's Hospital, Hanchuan, Hubei Province, China.

**Keywords:** coronary artery disease, long non-coding RNA, meta-analysis, polymorphism, protocol

## Abstract

**Background:**

Previous epidemiological studies displayed that long non-coding RNA (LncRNA) polymorphisms are associated with an increased risk of coronary artery disease, while the results are inconsistent. Therefore, we conducted a meta-analysis to more accurately determine the association between LncRNA polymorphism and the risk of coronary artery disease.

**Methods:**

PubMed, EmBase and Web of Science databases were searched, and the time to build the database was set until December 2020. The association between LncRNA polymorphism and the risk of coronary artery disease was collected and evaluated. Meta-analysis was performed by STATA 14.0 software, and the odds ratio and its 95% confidence interval (95%CI) were applied to estimate the association between LncRNA polymorphism and the risk of coronary artery disease.

**Results:**

The results of this meta-analysis will be submitted to a peer-reviewed journal for publication.

**Conclusion:**

This meta-analysis will summarize the relationship between LncRNA polymorphism and coronary disease risk.

**Ethics and dissemination:**

Ethical approval was not required for this study. The systematic review will be published in a peer-reviewed journal, presented at conferences, and shared on social media platforms. This review would be disseminated in a peer-reviewed journal or conference presentations.

**OSF REGISTRATION NUMBER:**

DOI 10.17605/OSF.IO/9XPHS.

## 1 Introduction

Coronary artery disease is a common cardiovascular disease, and is still one of the main causes of death worldwide.^[1,2]^ In developed countries such as the United States, it is the leading cause of death. In recent years, many studies confirmed that coronary artery disease has familial heredity through familial cluster analysis of coronary artery disease, and its genetic ability is predicted to be 50% to 60%.^[3]^ Early detection, early diagnosis and early treatment are the key for the improvement of the survival rate and the decrease of the mortality of coronary artery disease.^[4–6]^

In recent years, there was a new update in the research content of Genome-Wide Association Studies that is devoted to the analysis of the association between genes and complex diseases, and has made a major breakthrough.^[7–10]^ Mining a large number of gene polymorphic loci that is closely related to the risk of coronary artery disease will lay a foundation for future researches on the occurrence and development of coronary artery disease and genetic susceptibility to coronary artery disease. Early identification of gene polymorphic loci in susceptible populations is of vital significance in the treatment of unaffected and clinical prevention of coronary artery disease, because gene polymorphism is stably inherited in individuals and cannot be changed among many risk factors for coronary artery disease.

Long non-coding RNA (LncRNA) is the core of research in many fields such as genetics. In the past, people once thought that LncRNA was a non-coding gene, called “junk gene” that did not have the function of protein coding, so it was ignored. However, with the development of science and technology and the deepening of research, scholars generally believe that many kinds of cell life activities cannot be separated from the regulation of LncRNA. In recent years, new studies have reported that LncRNA plays an abnormal regulatory role in many complex diseases, including neoplastic diseases, ischemic stroke, Alzheimer's disease, cardiovascular diseases and so on.^[11–14]^

Many human diseases are associated with the abnormal expression of LncRNA. Some coronary heart disease-related genes and gene polymorphisms revealed that LncRNA polymorphism is correlated with sensitivity to coronary artery disease.^[15]^ LncRNA includes different transcriptional populations and is an important component of the gene regulatory network. It has many functions, including structural or transport functions, regulating cell cycle, differentiation, maintaining the integrity of cells and tissues, and regulating apoptosis.^[16]^ Although the mechanism of LncRNA in cardiac development and cardiovascular disease is not completely clear, a large number of studies have found some clues that can support the role of LncRNA in cardiovascular system.^[17–20]^

In addition, the abnormal expression of LncRNA plays an important role in the occurrence and development of coronary artery disease,^[21,22]^ which clearly indicates that LncRNA polymorphism can be used as a biomarker to evaluate the risk of coronary artery disease. Many studies have explored the relationship between LncRNA polymorphism and the risk of coronary artery disease. However, the results of these studies are not consistent.^[23–25]^ Therefore, we conducted a meta-analysis to examine the accurate correlation between LncRNA polymorphism and susceptibility to coronary artery disease.

## 2 Methods

### 2.1 Study registration

The protocol of this review was registered in OSF (OSF registration number: DOI 10.17605/OSF.IO/9XPHS). It was reported to follow the statement guidelines of preferred reporting items for systematic reviews and meta-analyses protocol.^[26]^

### 2.2 Inclusion criteria

Studies would be included in this meta-analysis based on the following criteria:

1) Types of studies: All case control studies associated with the susceptibility of LncRNA polymorphisms to coronary artery disease would be incorporated in our review. No restriction would be put on the publication date or status of the study.
2) Types of participants: Participants suffering from coronary artery disease will be included in the meta-analysis. Control subjects should be defined as without coronary artery disease or healthy individuals. No restrictions would be placed on age, sex, or country.
3) Data of the LncRNA polymorphism could be available on genotype distributions for the estimation of the odds ratio (OR) with its 95% confidence interval, or adequate data is provided to estimate the corresponding estimate effects (OR, 95% confidence interval).
4) Outcome: Coronary artery disease risk comparison.

### 2.3 Exclusion criteria

Studies would be excluded from the meta-analysis based on the following criteria: conference abstracts, case reports, unpublished articles, review papers, in vitro or animal studies, family-based studies, studies with insufficient data to calculate genotyping distribution, and repeated reports

### 2.4 Search strategy

Electronic searching would focus on databases of Pubmed, Web of Science, and Embase, with the temporal from the inception of database to December 2020. The combination of Medical Subject Headings alongside free terms would be used to hunt all the potentially eligible publications. The search strategy for PubMed is illustrated in Table 1, and the corresponding keywords would be used in other databases. Furthermore, we also supplements this search by manually searching the reference lists of related articles.

**Table 1:** Search strategy in PubMed database.

Number	Search terms
#1	Coronary Disease[MeSH]
#2	Coronary Heart Disease[Title/Abstract]
#3	Coronary Diseases[Title/Abstract]
#4	Coronary Heart Diseases[Title/Abstract]
#5	Disease, Coronary[Title/Abstract]
#6	Disease, Coronary Heart[Title/Abstract]
#7	Diseases, Coronary[Title/Abstract]
#8	Diseases, Coronary Heart[Title/Abstract]
#9	Heart Disease, Coronary[Title/Abstract]
#10	Heart Diseases, Coronary[Title/Abstract]
#11	Coronary Artery Disease[MeSH]
#12	Arteriosclerosis, Coronary[Title/Abstract]
#13	Atherosclerosis, Coronary[Title/Abstract]
#14	Coronary Arteriosclerosis[Title/Abstract]
#15	Coronary Atherosclerosis[Title/Abstract]
#16	Arterioscleroses, Coronary[Title/Abstract]
#17	Artery Disease, Coronary[Title/Abstract]
#18	Artery Diseases, Coronary[Title/Abstract]
#19	Atheroscleroses, Coronary[Title/Abstract]
#20	Coronary Arterioscleroses[Title/Abstract]
#21	Coronary Artery Diseases[Title/Abstract]
#22	Coronary Atheroscleroses[Title/Abstract]
#23	Disease, Coronary Artery[Title/Abstract]
#24	Diseases, Coronary Artery[Title/Abstract]
#25	or/1-24
#26	Long non-coding RNA [Title/Abstract]
#27	LncRNA[Title/Abstract]
#28	or/26-27
#29	polymorph∗[Title/Abstract]
#30	susceptibility[Title/Abstract]
#31	or/29-30
#32	#25 and #28 and #31

### 2.5 Data collection and analysis

#### 2.5.1 Selection of studies

**Figure 1:**
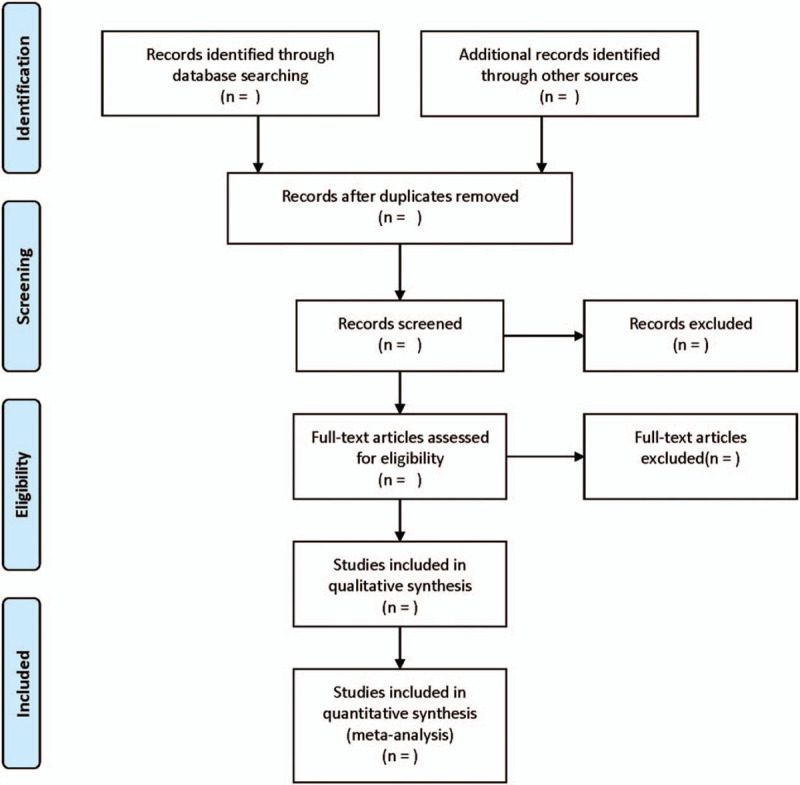
Flow diagram of study selection process.

The researchers of our team were trained professionally in advance on the purpose and process of the review. The two reviewers complete the screening process independently, and any differences are decided by the third reviewer. The screening process of the article includes reading the title, the abstract and the full text to determine whether it meets the inclusion criteria. The researchers record the reasons for excluding each study in light of the preferred reporting items for systematic reviews and meta-analysis guidelines and report the screening results. The flowchart is demonstrated in Figure 1.

#### 2.5.2 Data extraction

Each paper is comprehensively evaluated, and the data, including the year of publication, the surname of the first author, the nationality, country, average age, sex, number of subjects, detection method, LncRNA type, allele frequency and gene type distribution of LncRNA polymorphisms, is extracted independently by three researchers. Group discussions is conducted to resolve any disagreements in the extraction process. If the data for a paper is incomplete or unconvincing, we would try to contact the author via email. What's more, the Hardy–Weinberg equilibrium (HWE) of genotype distributions in the control group would also be examined.

#### 2.5.3 Study quality assessment

The quality of all the included studies is evaluated by 2 reviewers independently based on the Newcastle-Ottawa scale that is used to evaluate the quality of observational studies.^[27]^ Disagreement is reported and resolved by a third reviewer. Three broad perspectives of each study quality are scored: the selection of the study groups, the comparability of the case and control groups, and the determination of the exposure or outcome of interest in the studies. The Newcastle-Ottawa scale values arrange from 0 to 9. Studies with a score of 6 are considered to be of high quality.^[28]^

#### 2.5.4 Dealing with missing data

The reason for the loss of data in the period of data screening and extraction is identified here. We would attempt to contact the authors if the data of potential studies are insufficient, missing, or vague. These studies would be excluded only if the data are not available through the method described above.

#### 2.5.5 Statistical analysis

Statistical analyses were conducted by using Stata 14.0 (Stata Corporation, College Station, TX). The HWE for control subjects of each studies were evaluated by a chi-square test, and *P* < .05 was regarded as significant disequilibrium. The strength of association between LncRNA polymorphisms and susceptibility of cancer was assessed by computing the crude ORs with 95% confidence intervals. The pooled ORs were conducted for 4 genetic (allelic genetic model: T versus C; recessive genetic model: TT versus CT + CC; dominant genetic model: TT + CT versus CC; and additive model: TT versus CC. T and C represent the mutant allele and the wild-type allele, respectively). The significance of the pooled ORs is determined by *Z*-test, with *P* < .05 considered statistically significant. *x*^2^ test-based *Q* statistic and *I*^2^ would be applied to assess the overall heterogeneities. If *I*^2^ values < 50% and *P* > .1, heterogeneity is deemed to be low, and a fixed-effect model would be selected for data integration. Otherwise, a random-effect model could be used.

#### 2.5.6 Assessment of heterogeneity

Heterogeneity among the included studies will be evaluated by *I*^2^ statistic. A fixed-effects or random-effects model is utilized to measure pooled OR in the absence or presence of heterogeneity, respectively. When a set of studies exhibit an obvious heterogeneity, factors such as the characteristics of patients and the variation degree in exposure leading to the heterogeneity should be discussed. Subgroup analysis and sensitivity analysis would be conducted to explore potential sources of heterogeneity across studies when statistical heterogeneity is detected.

#### 2.5.7 Subgroup analysis

According to Up-regulated and down-regulated LncRNA, different ethnicity, and genotyping method, and so on, we carried out subgroup analyses of the relationships between LncRNA genetic polymorphisms and the risk of coronary artery disease.

#### 2.5.8 Sensitivity analysis

Through the study of large weight of elimination effect, the sensitivity analysis was performed to test the stability of the results of meta-analysis.

#### 2.5.9 Assessment of publication biases

The funnel plots will be used to examine the publication bias if there are > 10 eligible studies.^[29,30]^

#### 2.5.10 Grading the evidence quality

We utilize Grading of Recommendations Assessment, Development and Evaluation method to evaluate the evidence quality of the results obtained.^[31]^ The evaluation involves risk of bias exhibited by studies, the heterogeneity between groups, the estimate precision of effect, evidence directness, and publication risk of bias. The evidence quality is classified into 4 grades: high quality, moderate quality, low quality, and very low quality.

#### 2.5.11 Ethics and dissemination

The content of this article does not involve moral approval or ethical review and would be presented in print or at relevant conferences.

## 3 Discussion

Coronary artery disease is one of the major fatal diseases in the world.^[32]^ The disease is considered to be a complex multi-factor disease caused by the interaction of many environmental and genetic factors.^[33]^ Traditional risk factors are thought to be the cause of most coronary artery diseases. The percentage of 15%-20% of patients have no identified risk factors, which makes it impossible to prevent adverse cardiovascular events through appropriate treatment. Early identification and intervention strategies are essential to reduce the morbidity and mortality of coronary artery disease in high-risk groups. The determination of the genetic composition of diseases is an important field of cardiovascular disease research. With the rapid development of molecular technology and computer technology in recent years, many scholars have conducted in-depth and thorough researches on coronary artery disease-based susceptibility genes and their mutations. Genome-wide association studies have confirmed that genes are associated with susceptibility to coronary artery disease in different populations around the world.

Up to now, although many studies have focused on the relationship between LncRNA polymorphism and susceptibility to coronary artery disease, the accumulated evidence has not been systematically evaluated. In this study, we conducted a systematic review and meta-analysis of many research results to obtain more reliable risk association estimates, thus providing guidance for the prevention and treatment of coronary artery disease. The advantages of this study include following aspects: We included the latest literature for the exploration of heterogeneity, and tried to avoid post-group and subgroup analysis to improve the credibility of the results. We carried out a sensitivity analysis of each genetic model.

In conclusion, this study provides the latest evidence supporting LncRNA polymorphism and the susceptibility to coronary artery disease, and sheds light on strategies for the prevention and treatment of coronary artery disease.

## Author contributions

**Conceptualization:** Tao He, Bolin Wang.

**Data curation**: Bolin Wang and Zhihui Su.

**Formal analysis**: Zhihui Su.

**Funding acquisition:** Tao He.

**Investigation:** Zhihui Su.

**Methodology**: Zhihui Su.

**Project administration**: Tao He.

**Resources:** Lijun Wan.

**Software:** Lijun Wan.

**Supervision:** Lijun Wan, Tao He.

**Validation**: Lijun Wan.

**Visualization and software**: Bolin Wang and Zhihui Su.

**Writing – original draft**: Bolin Wang and Tao He.

**Writing – review & editing**: Bolin Wang and Tao He.
